# Rate of torque development and striatal shape in individuals with prodromal Huntington's disease

**DOI:** 10.1038/s41598-020-72042-2

**Published:** 2020-09-15

**Authors:** Travis Cruickshank, Alvaro Reyes, Timothy S. Pulverenti, Tim Rankin, Danielle M. Bartlett, Anthony J. Blazevich, Govinda Poudel, Mel Ziman, Gabriel S. Trajano

**Affiliations:** 1 School of Medical and Health Sciences, Edith Cowan University, 270 Joondalup Drive, Joondalup, Perth, WA 6027 Australia; 2 Exercise Medicine Research Institute, School of Medical and Health Sciences, Edith Cowan University, Joondalup, WA Australia; 3 Facultad de Ciencias de La Rehabilitacion, Universidad Andres Bello, Santiago, Chile; 4 Department of Physical Therapy, College of Staten Island, City University of New York, Staten Island, NY USA; 5 Centre for Exercise and Sports Science (CESSR), Edith Cowan University, Joondalup, WA Australia; 6 Mary Mackillop Institute for Health Research, Australian Catholic University, Melbourne, Australia; 7 School of Biomedical Science, University of Western Australia, Crawley, WA Australia; 8 School of Exercise and Nutrition Sciences, Faculty of Health, Queensland University of Technology (QUT), Brisbane, Australia

**Keywords:** Basal ganglia, Neurological disorders

## Abstract

The aim of the present study was to quantify explosive joint torque or the ability to develop joint torque rapidly, typically measured as the rate of torque development, in individuals with prodromal Huntington’s disease and healthy controls and its associations with measures of disease burden and striatal pathology. Twenty prodromal Huntington’s disease and 19 healthy control individuals volunteered for this study. Plantar flexor isometric rate of torque development values were evaluated using isokinetic dynamometry. Pathological changes in striatal shape were evaluated using magnetic resonance imaging. Disease burden was evaluated using the disease burden score and cytosine-adenine-guanine age product score. No statistical differences in the rate of torque development were observed between individuals with prodromal Huntington’s disease and healthy controls. However, significant associations were observed between the rate of torque development values and measures of disease burden (r = −0.42 to −0.69) and striatal pathology (r = 0.71–0.60) in individuals with prodromal Huntington’s disease. We found significant associations between lower rate of torque development values and greater striatal shape deflation and disease burden and striatal pathology in individuals with prodromal Huntington’s disease. While no significant differences in the rate of torque development were found between prodromal Huntington’s disease and healthy controls, the noted associations suggest that differences may emerge as the disease advances, which should be investigated longitudinally in future studies.

## Introduction

Motor impairments are a common and disabling aspect of Huntington’s disease (HD) that arise early and worsen gradually as the disease advances^1–3^. Reduced force steadiness and lower limb muscle strength are particularly apparent and contribute to postural instability, walking impairments and an overall decrease in functional independence^1,4–6^. Despite the severe impact of these impairments on functional independence, few studies have investigated the neuromuscular origins of motor impairments. Indeed, there has been no research on the capacity of individuals with HD to rapidly develop force, which may, at least in part, underlie early impairments in postural stability.

The rate of joint torque development (RTD) reflects to the ability of the muscles to produce high levels of force in a limited time period^7^. RTD is typically measured during explosive contractions in which individuals have to switch from complete muscle inactivity to maximal muscle activation in the shortest time possible^8^. When compared to measurements of maximal force, RTD is considered to be a more sensitive measure of neuromuscular deficits and has been shown to correlate with performances in activities of daily living, including walking, rising from a chair and postural stability in the elderly^9–12^. RTD has also been shown to be a sensitive predictor of motor symptom severity in individuals with Parkinson’s disease (PD)^13,14^ as well as upper limb function in people with multiple sclerosis (MS)^15^. These latter findings are relevant as individuals with PD and MS exhibit striatal degeneration, which is the principle neuropathological feature of HD and is a key structure involved in the regulation of movement.

Longstanding evidence suggests that the striatum is involved in inhibiting and facilitating movement^2,16^. The striatum achieves this through the activation of two molecularly distinct populations of medium spiny projection neurons: the indirect striatopallidal and direct striatonigral neurons. Activation of direct striatonigral neurons facilitates movement, whereas the activation of indirect striatopallidal neurons inhibits movement^17,18^. Preclinical and post-mortem data shows that both populations of neurons become dysfunctional and die throughout the course of HD, which is supported by neuroimaging studies in patients where the striatum shows early degeneration^2,3,19^. Dysfunction and loss of these neuronal populations is thought to underpin, at least in part, the onset of motor problems^20,21^ including the inability to rapidly generate movement, which is of fundamental importance to walking and maintaining postural stability. However, to date no study has examined whether RTD deficits exist and correlate with striatal integrity in individuals with HD.

The aims of the present study were therefore to compare RTD in individuals with prodromal HD (pro-HD) to that of healthy controls (HC), and then to examine the associations between RTD and striatal integrity and dynamic postural stability in individuals with pro-HD. It was hypothesised that RTD would be reduced in individuals with pro-HD and be associated with reduced striatal integrity and dynamic postural stability.

## Materials and methods

### Study approval and participant consent

All components of the study were performed in accordance with the Declaration of Helsinki. Ethical approval for this study was granted by the North Metropolitan Area Mental Health Service (NMAMHS) and Edith Cowan University Human Research Ethics Committees. Written and informed consent was provided by all participants prior to undertaking testing procedures.

### Participants

Twenty individuals with pro-HD and nineteen age- and sex-matched healthy controls (HC) were recruited for the study (Table 1). Individuals with pro-HD were recruited from the Neurosciences Unit of the North Metropolitan Area Mental Health Service (Perth, Western Australia). HC were recruited through public advertisement (radio, social media and flyer advertisements). Inclusion criteria were as follows: 1) a cytosine-adenine-guanine (CAG) repeat length ≥ 39, a total functional capacity score of 13, diagnostic confidence score ≤ 2 on the Unified Huntington’s Disease Rating Scale-Total Motor Score (UHDRS-TMS) and the ability to follow written or verbal instructions and provide written and informed consent. Exclusion criteria for both HD and HC were the presence of musculoskeletal, metabolic, endocrine, cardiovascular or sleep disorders or recent or longstanding substance abuse. Participants were screened for eligibility over the phone and in person. Disease burden score was calculated using the methods suggested by Penney et al.^22^ An index to estimate proximity to diagnosis at study entry was obtained using the CAG-age product (CAP) score. CAP score was calculated by multiplying the age at study entry by a scaling of the CAG repeat length as follows: CAP = (Age × (CAG – 33.66))/432.3326. CAP scores of < 1, 1 and > 1 indicate a 5-year diagnosis probability of < 0.5, 0.5 and > 0.5, respectively^23^.

**Table 1:** Physical and physiological characteristics of study groups.

Variable	pro-HD (n = 20)	HC (n = 19)	*p* value
Age (years)	43.30 (15.43)	43.72 (8.98)	0.458^a^
Sex (female/male)	13/7	13/5	0.632^b^
CAG repeat	43.40 (3.23)	–	–
Disease Burden Score	303.55 (89.26)	–	–
CAP score	0.87 (0.21)	–	–
UHDRS-TMS	7.30 (8.29)	–	–
Diagnostic Confidence Level (0/1/2)	11/5/4 0.61 (0.80)	–	–

*pro-HD* prodromal Huntington’s disease, *HC* healthy controls, *CAG* cytosine-adenine-guanine, *UHDRS-TMS* Unified Huntington’s Disease Rating Scale-Total Motor Score, *CAP score* CAG-age Product Scaled score.

^a^Independent *t*-test; ^b^Chi-square test.

### Study measures

#### Maximum voluntary contractions (MVCs)

Participants performed isometric plantarflexor maximal voluntary contractions (MVCs) in the seated position in an isokinetic dynamometer (Biodex 4, Biodex Medical Systems, NY, USA). Participants sat in the dynamometer chair with the hip joint slightly flexed (35°), the knee joint fully extended (0°), and the ankle joint of the right foot in a neutral position (0°) with the sole of the foot perpendicular to the shank, and the lateral malleolus of the fibula aligned to the centre of rotation of the dynamometer. Prior to testing, participants were asked to perform submaximal contractions of increasing intensity and MVCs as part of a warm-up. Following these warm-up procedures, participants performed 3—5 MVCs lasting between 3 and 5 s each with a 1-min rest interval between contractions. Visual feedback and strong verbal encouragement were provided during MVCs and participants were instructed to push the ankle attachment “as hard and fast as you can”. MVC trials were repeated until there was a difference of less than 5% in maximal torque. Ankle joint torque was sampled at 2000 Hz and recorded on a personal computer running LabChart Software (ADInstruments, NSW, Australia) using a 16-bit analogue-to-digital converter (PowerLab 16/35, ADInstruments, NSW, Australia).

#### Rate of torque development (RTD)

**Figure 1:**
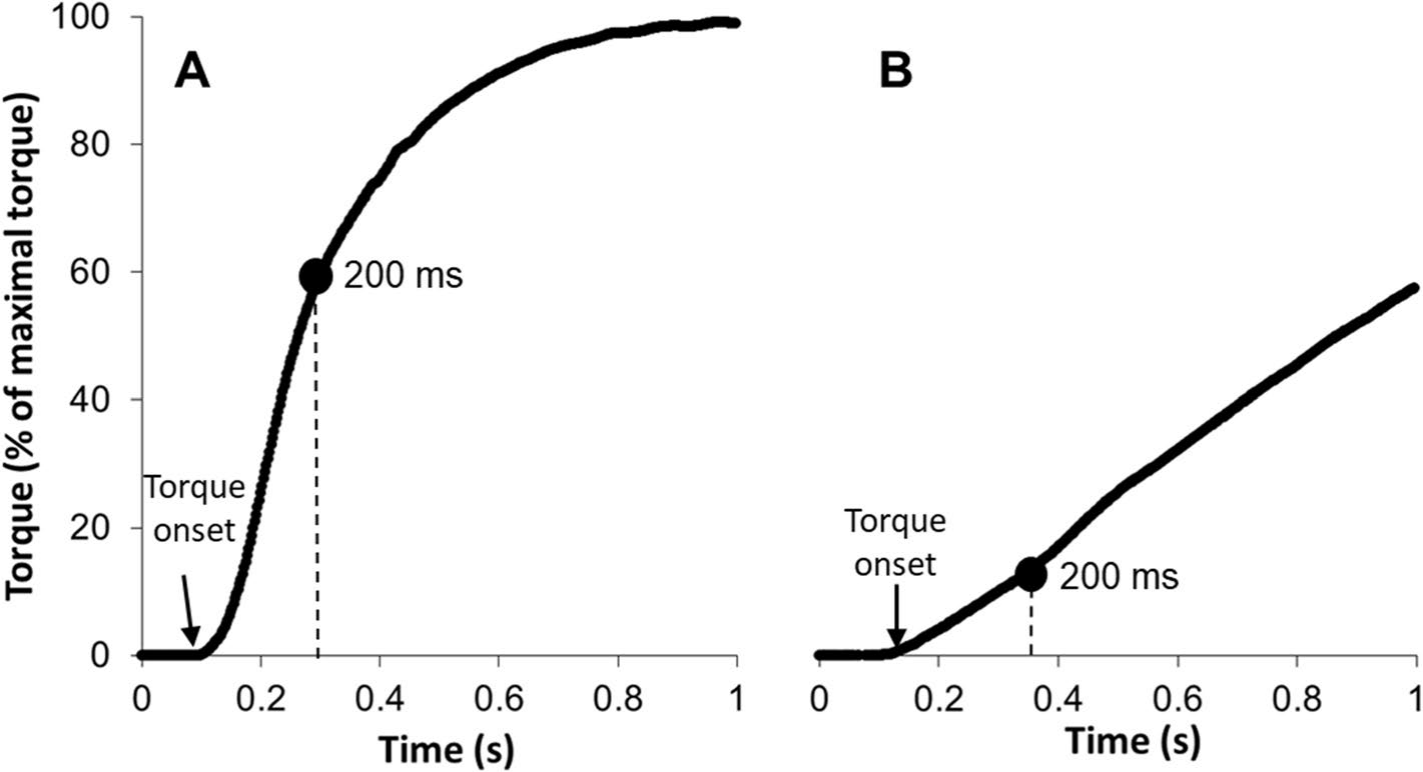
Example of measurement of torque onset and rate of torque development (RTD). RTD was measured as the average slope from torque onset to 200 ms. Panel A shows an individual with pro-HD with high RTD and panel B shows an individual with pro-HD with low RTD.

The RTD was measured using the unfiltered torque traces during the maximal voluntary plantar flexor contractions. The RTD was calculated as the average change in torque per time interval from the torque onset to 200 ms (see Fig. 1). Torque onset was defined as the last point before the torque trace deflected above the range of baseline, determined visually^10^. The two highest values obtained for each time interval were selected and averaged. In order to test the intrinsic capacity of the muscle to develop rapid force by accounting for peak force capacity, the relative RTD was calculated by normalising the RTD values to MVC peak torque. This normalisation allowed for comparisons between subjects of different strength capacity.

#### Limits of stability test (LOS)

The LOS test was used to examine dynamic postural stability. The LOS has demonstrated sensitivity in individuals with premanifest HD previously^5^. Participants were asked to stand comfortably on the force plates and focus on a computer screen placed at eye level to provide visual feedback. Participants were instructed to transfer their weight as quickly and accurately as possible toward one of eight targets displayed on the screen without moving their feet. Targets are located in forward (FW), forward-right (FWRT), right (RT), backward-right (BWRT), backward (BW), backward-left (BWLT), left (LT) and forward-left (FWLT) directions throughout the test. The mean of the eight trials completed for each target was calculated for five LOS dependent variables: reaction time (RT), movement velocity (MV), endpoint excursion (EE), maximum excursion (ME) and directional control (DC). RT represents the time between the presentation of the visual signal to move and the beginning of the participant´s movement, MV is the average speed (degrees/seconds) of the COG in a specific direction, EE is the distance travelled by the COG in the first movement, ME is the farthest distance travelled by the COG and DC is the difference between the amount of movement in the intended direction and the amount of movement the COG deviated form from a straight line. For RT, a higher score represents a poorer performance, while for MV, EE, ME and DC, a higher score represents a better performance. RT and MV are expressed as absolute values and EE, ME and DC are expressed as a percentage of maximum theoretical LOS values as determined by Nashner et al. Balance Manager® Systems (2011)^19^. Results are presented using the average of the 8 directions (targets) for each variable (Table 2) and separated by direction (Fig. 1).

**Table 2:** Median, 25th (p25), 75th (p75) percentiles and *p* values for between-group comparisons in RTD, limits of stability and striatal shape between individuals with pro-HD and HC.

	Pro-HD Median (p25; p75)	HC Median (p25; p75)	*p* value
Relative RTD _%MVC/s_	1.41 [0.83; 2.59]	1.95 [1.21; 2.59]	0.159
Absolute RTD _Nm/s_	162.05 [69.16; 288.00]	178.83 [74.98; 231.08]	0.754
Left caudate _a.u_	−0.11 [−0.57; 0.07]	0.47 [−0.05; 0.63]	0.015
Right caudate _a.u_	−0.09 [−0.60; 0.08]	0.29 [0.01; 0.56]	0.001
Left putamen _a.u_	−0.06 [−0.53; 0.20]	0.17 [0.02; 0.31]	0.037
Right putamen _a.u_	−0.16 [−0.48; 0.10]	0.20 [0.07; 0.39]	0.001
Reaction time (ms)	0.79 [0.56; 0.9]	0.92 [0.74; 0.98]	0.224
Movement velocity (deg/s)	3.13 [2.46; 3.93]	3.32 [2.68; 3.71]	0.475
End point Excursion (%)	55.25 [42.00; 61.75]	72.06 [64.87; 78.25]	0.000
Maximum excursion (%)	77.12 [66.12; 85.75]	90.37 [85.00; 93.12]	0.000
Directional control (%)	80.75 [78.37; 83.25]	74.37 [55.25; 81.37]	0.010

*pro-HD* prodromal Huntington’s disease, *HC* healthy controls, *RTD* rate of torque development, *MVC* maximum voluntary contractions. Data are presented as median [Q1; Q3]. Results were considered significant at *p* < 0.05.

### Magnetic resonance imaging (MRI)

T_1_-weighted structural brain MRI images were acquired from each participant using 3 T MRI (GE Healthcare Discovery MR750W 3 T, Milwaukee, USA). Images were acquired with a 24-channel head coil using an IR-SPGR sequence (TA = 9 m 59 s, TR = 3 s, TE = 3.1 ms, TI = 400 ms, flip angle = 11°, field of view = 256 mm × 256 mm, image matrix = 256 × 256, 1 mm^3^ isotropic voxels).

### Shape analysis

T_1_-weighted MRI data were analysed for caudate and putamen shape. This approach looks at how a structure may differ in shape (e.g. deflation) between two groups (e.g., pro-HD and HC) by directly comparing the shape meshes on a vertex by vertex basis. FSL-integrated registration and segmentation toolbox (FSL-FIRST) software, a model-based registration/segmentation tool, was used for segmentation of the sub-cortical structures. The caudate nucleus and putamen were segmented from the T_1_-weighted MR images. The deformable surfaces of the putamen and caudate were used to automatically parameterise the volumetric labels in terms of meshes. The normalised intensities along the surface of meshes were sampled and modelled, with the shape and appearance model based on multivariate Gaussian assumptions^24^. The segmentations were manually checked to ensure quality. To investigate the association between RTD and caudate and putamen shape, vertex-wise shape values for each region were extracted for use in correlation analyses.

### Statistical analysis

Sample size was calculated based on an estimated Pearson correlation coefficient of at least 0.6 between measures of RTD and striatal shape. Using a bilateral test, an alpha level of 0.05, a statistical power of 0.8, it was estimated a total sample size of at least 19 participants per group would be sufficient to detect differences.

Descriptive data are presented as mean and standard deviation for normally distributed data and as median and interquartile range for non-normal data, as determined using the Shapiro–Wilk test. An independent t-test was used to test age differences between groups and a Chi-square test was used to test for sex differences. Spearman’s correlation coefficients were computed to examine the association between RTD and measures of disease burden. U Mann–Whitney tests were used to compare RTD, limits of stability and both caudate and putamen shape between groups. Pearson’s correlations were computed to evaluate associations between RTD, limits of stability and both caudate and putamen shape. Correlation coefficients were considered weak, moderate or strong at 0.3, 0.5 and 0.7, respectively. Test–retest reliability of RTD measurements were evaluated by intraclass correlation coefficients (ICC). Statistical significance was set at *p* ≤ 0.05. Statistical analyses were performed using STATA version 15.1 (StataCorp. 2017. *Stata Statistical Software: Release 15*. College Station, TX: StataCorp LLC).

## Results

Thirty-nine participants completed all experimental procedures required for the study. Clinical and demographic data are presented in Table 1. No significant differences were observed between groups for age or sex.

### Reliability and variability analysis

Healthy controls had higher ICC values for absolute RTD (0.96 [95% CI 0.94 – 0.99]) and relative RTD (0.87 [95% CI 0.77 – 0.98]) than individuals with pro-HD for absolute RTD (0.92 [95% CI 0.85 – 0.98]) and relative RTD (0.75 [95% CI 0.56 – 0.94]). Coefficient of variation (%) of ICC values were higher in individuals with pro-HD for absolute RTD (3.6%) and relative RTD (12.9%) than in HC for absolute RTD (1.4%) and relative RTD (6.0%). Standard error of measurement (SEM) was higher in individuals with pro-HD for absolute RTD (16.9) and relative RTD (0.24) than HC for absolute RTD (4.3) and for relative RTD (0.12).

### Between-group analysis

**Figure 2:**
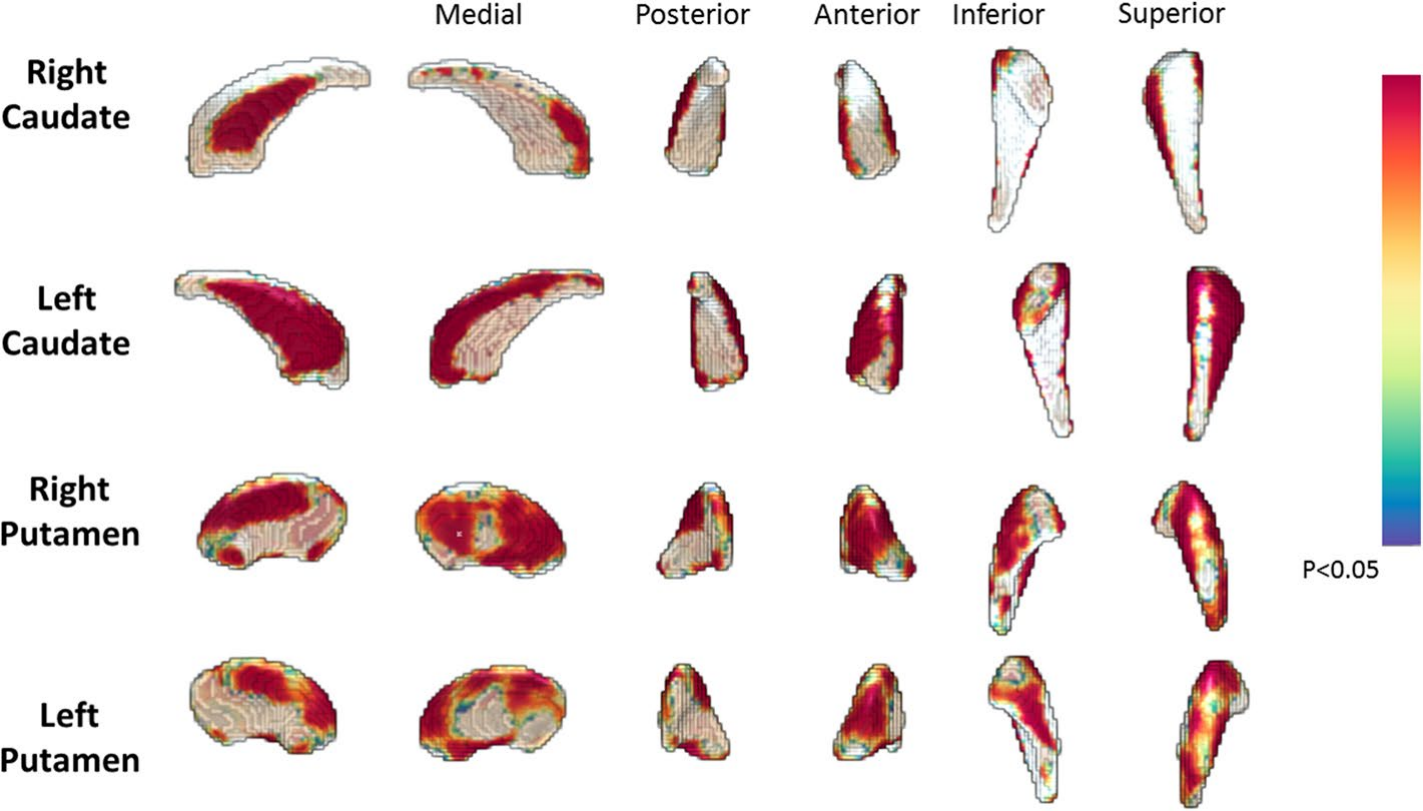
Vertex-wise shape differences between individuals with prodromal HD and healthy controls. The differences were significant at *p* < 0.05 (corrected). The colour bar represents the percentage of atrophy at a specific vertex in the disease group relative to the control group.

Descriptive statistics and Mann–Whitney U test results for differences in RTD, limits of stability and striatal shape between pro-HD and HC are presented in Table 2. No between-group differences were observed for RTD. Significant reduced scores were detected for endpoint excursion, maximum excursion and directional control in individuals with prodromal HD compared to HC. Figure 2 shows cross-sectional shape differences between individuals with prodromal HD and HC (*p* < 0.05). Widespread differences in caudate and putamen shape were observed, with significantly greater deflation in pro-HD than HC.

### Association between the RTD and striatal shape, limits of stability and measures of disease burden

Table 3 shows associations between RTD, limits of stability and putamen and caudate shape in pro-HD and HC. Positive correlations were observed between RTD and striatum shape and endpoint excursion outcomes in pro-HD but not HC. A negative correlation was also observed between RTD and UHDRS-TMS in pro-HD but not HC.

**Table 3:** Correlation coefficients for associations between striatal shape, limits of stability and RTD in individuals with pro-HD and HC (Pearson’s) and associations between relative RTD and measures of disease burden (Spearman’s).

	Relative rate of torque development	
	Pro-HD	HC
Left caudate	0.71 (0.000)	− 0.07 (0.784)
Right caudate	0.60 (0.004)	− 0.29 (0.238)
Left putamen	0.66 (0.001)	− 0.11 (0.654)
Right putamen	0.65 (0.002)	− 0.28 (0.244)
Reaction time (ms)	− 0.00 (0.996)	− 0.14 (0.553)
Movement velocity (deg/s)	0.01 (0.946)	0.34 (0.161)
End point Excursion (%)	0.49 (0.032)	− 0.05 (0.820)
Maximum excursion (%)	0.40 (0.087)	− 0.05 (0.843)
Directional control (%)	0.43 (0.060)	0.01 (0.950)
Disease burden score	− 0.42 (0.053)	–
CAP score	− 0.37 (0.094)	–
UHDRS-TMS	− 0.69 (0.000)	–

*pro-HD* prodromal Huntington’s disease, *HC* healthy controls, *RTD* rate of torque development, *UHDRS-TMS* Unified Huntington’s Disease Rating Scale-Total Motor Score, *CAP score* CAG-Age Product Scaled score, *RTD* rate of torque development. Data are presented as correlation coefficients (*p* value). Results were considered significant at *p* < 0.05.

### Association between the RTD and measures of disease burden

Moderate to high significant, negative correlations were detected between UHDRS-TMS, CAP score, and disease burden score and RTD in individuals pro-HD (Table 3).

## Discussion

This study evaluated, for the first time, RTD and its association with striatal pathology, postural stability and clinical disease outcomes in individuals with pro-HD and HC. The data revealed no differences in RTD between pro-HD and HC. However, evaluation of the relationships between RTD and measures of striatal pathology, postural stability, and clinical disease outcomes revealed that greater striatal pathology, postural instability and disease burden was associated with reduced RTD in pro-HD.

Contrary to our hypothesis, our results revealed no systematic differences in relative or absolute RTD between HC and individuals with pro-HD. This suggests that explosive strength capacity is largely preserved in individuals with pro-HD. We hypothesised that individuals with pro-HD would have a reduced capacity to produce high RTD compared to HC, particularly considering the hallmark striatal degeneration and impairments in motor function in HD. However, it is important to note that a large range of values were evident in the HD group, with some individuals displaying greater explosive strength while others presented with very low explosive strength. This greater variability in the pro-HD group is in line with previous findings by Busse et al.^25^ who revealed greater variability for ankle dorsiflexion muscle strength in individuals with manifest HD than in HC. Together, these findings highlight the inherent variability in motor features across individuals with pro-HD, which has been well-documented in previous epidemiological studies and is presumed to relate to differences in striatal pathology, genetic burden, lifestyle and genetic modifiers, which could be the subject of future investigations.

Consistent with previous studies, significant differences in striatal shape metrics were observed between individuals with pro-HD and HC^26,27^. In particular, individuals with pro-HD displayed significant shape deflation in comparison with HC that was more evident for the right hemispheres of the putamen and caudate than for the left. Interestingly, striatal pathology was associated with RTD in individuals with pro-HD but not HC. To our knowledge, this is the first study to report an association between striatal shape and RTD in individuals with pro-HD. The nature of this association is not yet known, however evidence from preclinical and healthy ageing studies suggests that the striatum is strongly involved in the initiation of movement via direct neuronal pathways^28^. This is particularly relevant to our findings and suggests that striatal degeneration may mediate gradual changes in RTD in individuals with HD, which may become more evident as the disease advances. However, this is a tentative supposition, particularly considering that no systematic differences in RTD were observed between the pro-HD and HC group. There is, therefore, a need to evaluate the observed association between RTD and striatal degeneration longitudinally.

In line with our expectations, significant associations were observed between RTD and postural instability in individuals with pro-HD. Indeed, we observed a significant association between RTD and endpoint excursion. This is the first paper to our knowledge to report a link between RTD and endpoint excursion in individuals with pro-HD. This finding was not unexpected, as previous studies in healthy individuals have noted associations between RTD and maximum recoverable lean angle^29–32^. There is also a wealth of evidence showing that rapid force development in the lower limbs is required for anticipatory postural adjustments^33^. While somewhat expected, this finding has important implications with respect to the management of postural stability outcomes in individuals with HD. Indeed, it highlights the importance of incorporating RTD training in rehabilitation programs aimed at preserving postural stability in individuals with HD. This association nevertheless needs to be explored longitudinally.

Consistent with our expectations, significant associations were observed between RTD and measures of disease burden. Our results align with early findings by Busse et al.^25^, who reported significant correlations between maximum voluntary muscle strength using hand-held dynamometry and motor symptom severity in individuals with manifest HD. These findings are of interest and suggest that individuals with greater impairments in muscle function have more severe motor symptoms. The observed associations nevertheless need to be validated in larger cohorts of individuals with HD and explored longitudinally. If confirmed in future studies, it raises the interesting possibility of utilising rehabilitation interventions, including multidisciplinary rehabilitation, which has been previously shown to improve muscle strength in HD^34^, to combat changes in RTD and motor impairments.

Several limitations should be acknowledged. The present study was a cross-sectional investigation and therefore did not allow conclusions to be drawn with respect to changes in RTD and its associations with striatal pathology and measures of disease burden over time. The present study also included a small sample size, which makes generalisation of these findings difficult. Finally, this study only included individuals in the prodromal stage of the disease. It is possible that RTD deficits may present in the manifest stages of the disease. Subsequent studies are therefore needed to explore RTD in individuals with manifest HD.

Despite these limitations, the present paper reveals novel findings. In particular, RTD does not seem to be systematically impacted in the early stages of HD, although it is strongly associated with striatal shape as well as measures of postural stability and disease burden in individuals with pro-HD. These latter results suggest that RTD may deteriorate as the disease advances and contribute to postural instability over time. There is a fundamental need to explore RTD changes longitudinally as well as in manifest stages of the disease.
